# Gender differences in leukemia outcomes based on health care expenditures using estimates from the GLOBOCAN 2020

**DOI:** 10.1186/s13690-023-01154-8

**Published:** 2023-08-21

**Authors:** Maedeh Amini, Rajesh Sharma, Chinmay Jani

**Affiliations:** 1 Basic and Molecular Epidemiology of Gastrointestinal Disorders Research Center, Research Institute for Gastroenterology and Liver Diseases, Shahid Beheshti University of Medical Sciences, Tehran, Iran; 2 Humanities and Social Sciences, National Institute of Technology Kurukshetra, Kurukshetra, India; 3 Mount Aubrun Hospital, Cambridge, MA USA; 4 Harvard Medical School, Boston, MA USA

**Keywords:** Leukemia, Incidence, Mortality, Mortality-to-incidence ratio, GLOBOCAN 2020

## Abstract

**Background:**

Leukemia contributes significantly to the global cancer burden. Due to the importance of evaluating improvements in leukemia outcomes, the current study aimed to examine the variations in mortality-to-incidence ratio (MIR) between genders and association of MIR with the health expenditures in selected countries.

**Methods:**

The leukemia incidence and mortality rates were extracted from the GLOBOCAN 2020 database. In total, 56 countries were included based on the data quality reports and the exclusion of missing data. The associations of MIR and changes in MIR over time ($\updelta$MIR) with the human development index (HDI), current health expenditure (CHE) per capita, and current health expenditure as a percentage of gross domestic product (CHE/GDP) were investigated using Spearman’s rank correlation coefficient.

**Results:**

In 2020, an estimated 474,519 new cases of leukemia were diagnosed globally, and 311,594 deaths occurred due to the disease. Male patients exhibited a higher incidence and mortality of leukemia compared to females on a global scale. Our analysis revealed that the MIRs were the highest and lowest in Egypt (0.79) and the United States (0.29), respectively. Remarkably, countries with greater HDI, higher CHE per capita, and a higher CHE/GDP tended to have lower MIR in both genders and within gender-specific subgroups. The δMIR demonstrated a significant negative correlation with HDI and CHE per capita, whereas no significant associations were observed among female patients for CHE/GDP. Besides, all three indicators showed trends towards negative correlations with δMIR among males, though these trends were not statistically significant (*p*>0.05).

**Conclusions:**

Generally, leukemia MIRs tended to be most favorable (i.e., lower) in countries with high HDI and high health expenditure. The gender differences observed in leukemia outcomes may reflect the potential influence of social, material, behavioral, and biological factors.

**Supplementary Information:**

The online version contains supplementary material available at 10.1186/s13690-023-01154-8.

**Table:** 

**Text box 1. Contributions to the literature**
• Research has illuminated that ASR-based δMIR had positive values in several countries, which may suggest an improvement in detection and management of leukemia outcomes.
• The significant correlation between ASR-based MIR and healthcare evaluation indicators supported the conclusion that there are health care disparities among various countries.
• A gender inequality was evident for the correlation of MIR with CHE per capita and CHE/GDP across countries, confirming the impact of cancer care disparities in the prognosis of leukemia between genders.

## Background

Leukemia is a group of cancers that arise from abnormal cells derived from hematopoietic tissues within the body, exhibiting poor differential and aggressiveness [1]. Estimates from GLOBOCAN 2020 indicated that leukemia ranked as the 11^th^ leading cause of cancer-related mortality worldwide, accounting for nearly 4.7% (466,003) of all cancer-related deaths. Besides, leukemia was diagnosed as the 16^th^ most commonly occurring cancer, with over 495,000 new cases reported in 2020 (2.6%) [2]. Examining global trends, gradual decreasing trends were seen for both incidence and related deaths during the period 1990–2019. Nevertheless, it has been noticed that the age-standardized incidence rate (ASIR), age-standardized mortality rate (ASMR), incidence cases, and deaths related to leukemia per year are predicted to rise untill 2030 at the global level [3, 4]. Hence, it is imperative to allocate health resources in order to enhance leukemia prevention and control.

The incidence and mortality rates of leukemia have varied across regions and countries, which most likely reflect changes and differences in multiple risk factors, disease detection, diagnostic practices, treatment, healthcare infrastructure, disparities in the distribution of cancer cases, and complex etiology [5–11]. For instance, the ASMR reveled a remarkable downward trend in high-income geographic regions until 2017, while the upward trends were detected in Andean Latin America and East Asia [7]. Furthermore, the decreasing trend in ASIR was most pronounced in low-middle- and middle-sociodemographic index regions from 1990–2019 [3]. These observations confirm the requirement to examine the disparities in leukemia burden and healthcare systems among different nations.

The main clinical outcome for cancer treatment is typically measured by the 5-year survival rate, and partially by the mortality-to-incidence ratio (MIR). The MIR has been identified as a valuable indicator for assessing disparities in cancer screening, healthcare systems quality, and the long-term effectiveness of cancer control and surveillance programs. It's worth noting that some previous epidemiological studies have indicated that the MIR can be applied to determine whether a country experiences higher or lower mortality compared to its incidence. In countries where actual data on cancer survival rates is lacking, it can serve as a useful proxy to reflect relative survival rates among nations [6, 12, 13]. A prior study using the Global Burden of Disease (GBD) 2019 database demonstrated that the decline in leukemia incidence and mortality rates in developed regions was more significant than in developing regions [3]. Nevertheless, there is a scarcity of studies evaluating the progress in leukemia management outcomes over time and its correlation with country-level health disparities.

Notably, gender variations have a significant impact on cancer, resulting in incidence rates being up to 20% greater and mortality rates up to 40% higher among males [14]. A collection of previous studies has clarified that the notable difference between sexes is related to many factors, namely awareness, treatment, healthcare use, disease control rate, time of diagnosis, occupational exposure, and differences in overall survival [15–17]. In terms of leukemia, the male population exhibited higher rates of incidence and mortality compared to females, highlighting the impact of biologic and epidemiologic factors [1, 4, 10, 11, 18, 19]. Disparities in healthcare and socioeconomic factors among nations could contribute to certain aspects of leukemia risk factors. To explore gender differences in this context, we have implemented the relative MIR of leukemia (MIR of female to MIR of male), which has not previously been undertaken in existing studies. In general, the objectives of the present study are as follows. Firstly, to describe the incidence, mortality rates, and relative MIR of leukemia, thereby enabling a comparison of continental and regional variations; all of which is based on data from the GLOBOCAN 2020 database. Secondly, we provided the incidence and mortality rates (per 100,000), as well as MIR due to leukemia, human development index (HDI) values, current health expenditure (CHE) per capita, and CHE as a percentage of gross domestic product (CHE/GDP) for selected countries. Furthermore, the delta-mortality-to-incidence ratio ($\updelta$MIR) was determined for each country by subtracting the MIR values of GLOBOCAN 2020 from GLOBOCAN 2012. This indicator can help assess changes in MIR over time and improvements in clinical outcomes among the selected countries. Lastly, the correlations of MIR and $\updelta$MIR with HDI, CHE, and CHE/GDP were examined, providing valuable insights for health planning and research activities.

## Methods

### Data sources and variables

Our analysis was carried out using leukemia incidence and mortality data (ICD-10 codes C91-C95) sourced from the publicly available cancer database GLOBOCAN 2020, which is maintained by the International Agency for Research on Cancer of the World Health Organization (WHO) [2] (http://gco.iarc.fr/). Briefly, cancer incidence and mortality rates for 2020 were estimated by sex and across 18 age groups (0–4, 5–9, 10–14, 15–19, …, 75–79, 80–84, 85 and over) for the 185 countries or territories worldwide with populations exceeding 150,000 inhabitants in the same year. Reports are presented for 38 cancer sites or cancer types, as defined by the 10th edition of the International Classification of Diseases (ICD-10, version 2014), and for all cancers combined. The sources of data, as well as the methods hierarchy utilized in compiling the cancer estimates, have been described in detail elsewhere [20]. In the current study, the number, crude rate, and age standardized rate for leukemia were extracted by gender, continent, and country.

The inclusion criteria for selecting countries for this study encompassed the availability/quality of cancer incidence and mortality information by gender. Moreover, countries were excluded according to the data quality report from GLOBOCAN (*N* = 121), instances of missing data (*N* = 3), and outliers for MIR/ $\delta$MIR (*N* = 5). Consequently, a total of 56 countries fulfilled the inclusion criteria for data quality and were included in the final analysis. On the other hand, data relating to health expenditures, CHE per capita, and CHE/GDP were obtained from the World Health Statistics of WHO (https://www.who.int/gho/publications/world_health_statistics/en/). These indicators play a pivotal role in assessing healthcare disparity among different countries and contribute to our comprehension of the influence of MIR on healthcare systems [21].

The incidence and mortality of leukemia may be influenced by socio-demographic variables. One of the indicators that allows for comparing countries with respect to these key aspects is the HDI. The United Nations Development Programme described the HDI as a measure of average achievement in three key dimensions of human development: a long and healthy life, being knowledgeable, and having a decent standard of living. The HDI comprises education, estimated as the expected years of schooling and average years of schooling; income or standard of living measured by gross national income per capita; and population health based on life expectancy at birth (in years) [22]. The HDI data was obtained from the Human Development Report Office of the United Nations Development Programme (http://hdr.undp.org/en). Based on this indicator, the countries were classified into four groups: low (0.35–0.54), medium (0.55–0.69), high (0.70–0.79), and very high (0.80–1.00) [23].

The MIR is defined as the ratio of the crude rate (CR) of mortality to the CR of incidence. This indicator can identify whether a country has a higher mortality than might be expected according to its incidence [24]. The age-standardized rate (ASR)-based $\delta$MIR is also defined as the difference between the ASR- based MIR in 2012 and 2020 (ASR-based $\updelta$MIR = ASR-based MIR [in 2012] – ASR-based MIR [in 2020]). This index provides information about changes in MIR between these two years. Importantly, a positive value for ASR-based $\updelta$MIR may suggest an improvement in the detection and management of leukemia outcomes [25]. Additionally, we examined the relative MIR, defined as the ratio of the MIR for females to the MIR males.

### Statistical analysis

The associations among MIR, $\updelta$MIR, and other indicators (including HDI, CHE per capita, and CHE/GDP percentage) across selected countries, stratified by gender, were determined using Spearman’s rank correlation coefficient. It is important to emphasize that significant correlations highlight health care disparities among different countries. All statistical analyses and visualizations were implemented using SPSS version 21.0 (IBM SPSS Inc, Amonk, NY, USA) and R software version 4.3.0. A p-value of less than 0.05 from a two-sided test was regarded as statistically significant.

## Results

### Epidemiology of leukemia outcomes across regions

**Fig. 1:**
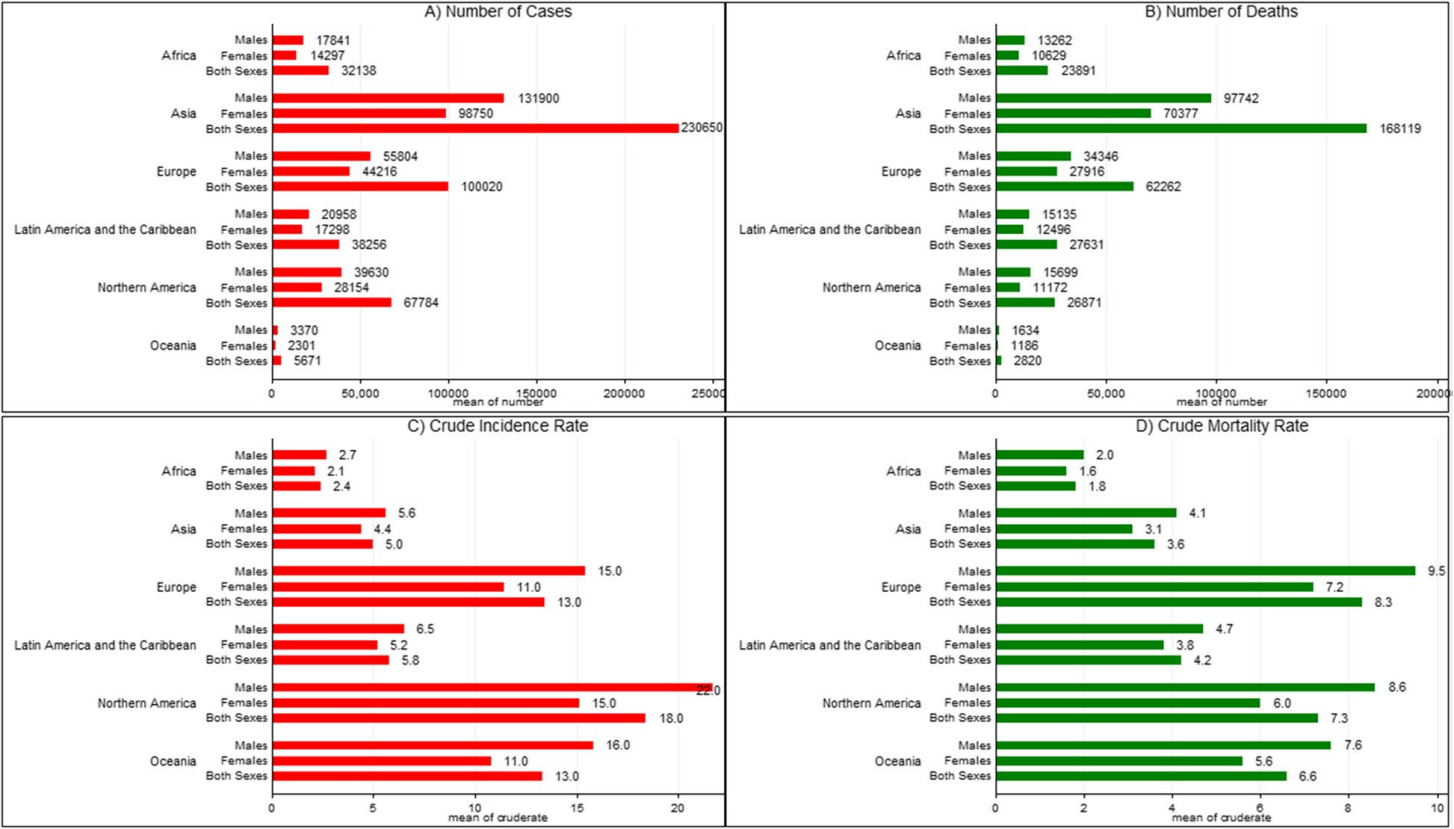
Incidence and mortality from leukemia in 2020 for each continent by gender

Overall, in 2020, there were 474,519 new cases of leukemia and 311,594 deaths attributed to the disease. The incidence of new cases and deaths was more pronounced among males globally compared to females (269,503 vs. 205,016; 177,818 vs. 133,776, respectively). Similar patterns were also observed in the CRs of incidence (6.9/100,000 in males vs. 5.3/100,000 in females) and mortality (4.5/100,000 in males vs. 3.5/100,000 in females) worldwide. The cases and CRs of incidence and mortality, along with the MIR, for the total population, females, and males across different regions and continents, are provided in Table 1. Among the various continents, Asia exhibited the highest numbers of new cases (230,650) and deaths (168,119) for both genders; whilst the lowest numbers were observed in Oceania (5671 new cases and 2820 deaths, respectively). Northern America had the highest CR of incidence (total: 18.4/100,000; female: 15.1/100,000; male: 21.7/100,000), whereas the lowest CR of incidence was recorded in Africa (total: 2.4/100,000; female: 2.1/100,000; male: 2.7/100,000). Furthermore, we found that Europe and Africa, had the highest and lowest CR of mortality, respectively. By WHO regions, the Western Pacific region had the highest absolute numbers of new cases and deaths for both sexes. In contrast, Africa had the lowest numbers of incidence and mortality. Moreover, Europe had the highest CR of incidence (total: 11.9/100,000; female: 10.3; male: 13.7), as well as the highest CR of mortality (total: 7.5/100,000; female: 6.5/100,000; male: 8.6/100,000), followed by the Americas (Fig. 1).

**Table 1:** Summary of leukemia incidence, mortality, and mortality-to-incidence ratio by gender for the regions

Region	Incidence, number			Mortality, number			Incidence, crude rate			Mortality, crude rate			MIR			
	Total	Female	Male	Total	Female	Male	Total	Female	Male	Total	Female	Male	Total	Female	Male	Relative MIR
Global	474,519	205,016	269,503	311,594	133,776	177,818	6.1	5.3	6.9	4	3.5	4.5	0.66	0.66	0.65	1.01
**Continent**																
Africa	32,138	14,297	17,841	23,891	10,629	13,262	2.4	2.1	2.7	1.8	1.6	2	0.75	0.76	0.74	1.03
Latin America and Caribbean	38,256	17,298	20,958	27,631	12,496	15,135	5.8	5.2	6.5	4.2	3.8	4.7	0.72	0.73	0.72	1.01
Northern America	67,784	28,154	39,630	26,871	11,172	15,699	18.4	15.1	21.7	7.3	6	8.6	0.40	0.40	0.40	1
Asia	230,650	98,750	131,900	168,119	70,377	97,742	5	4.4	5.6	3.6	3.1	4.1	0.72	0.70	0.73	0.95
Europe	100,020	44,216	55,804	62,262	27,916	34,346	13.4	11.4	15.4	8.3	7.2	9.5	0.62	0.63	0.62	1.02
Oceania	5,671	2,301	3,370	2,820	1,186	1,634	13.3	10.8	15.8	6.6	5.6	7.6	0.50	0.52	0.48	1.08
**WHO region**																
Western Pacific	126,250	54,816	71,434	89,528	38,025	51,503	6.4	5.7	7.1	4.6	3.9	5.2	0.72	0.68	0.73	0.93
Europe	111,438	49,339	62,099	70,385	31,465	38,920	11.9	10.3	13.7	7.5	6.5	8.6	0.63	0.63	0.63	1
Americas	106,040	45,452	60,588	54,502	23,668	30,834	10.4	8.8	12	5.3	4.6	6.1	0.51	0.52	0.51	1.02
South-East Asia	75,360	31,438	43,922	55,997	22,825	33,172	3.7	3.2	4.2	2.8	2.3	3.2	0.76	0.72	0.76	0.95
East Mediterranean	32,938	14,015	18,923	24,465	10,375	14,090	4.5	4	5	3.3	2.9	3.7	0.73	0.72	0.74	0.97
Africa	22,385	9,909	12,476	16,652	7,387	9,265	2	1.8	2.2	1.5	1.3	1.7	0.75	0.72	0.77	0.93
**Development index**																
Very high	205,780	88,585	117,195	114,133	49,404	64,729	13.2	11.2	15.2	7.3	6.2	8.4	0.55	0.55	0.55	1
High	167,149	72,513	94,636	121,631	51,977	69,654	5.7	5	6.4	4.2	3.6	4.7	0.74	0.72	0.73	0.99
Medium	80,695	34,655	46,040	60,031	25,373	34,658	3.5	3.1	3.9	2.6	2.2	2.9	0.74	0.71	0.74	0.96
Low	20,690	9,173	11,517	15,670	6968	8702	2.1	1.9	2.3	1.6	1.4	1.8	0.76	0.74	0.78	0.95

*WHO* world health organization; *MIR* mortality-to-incidence ratio

Comparing the HDI quintile groups, it was revealed that the region with a very high HDI region had the highest numbers of new cases and CRs of incidence (total: 13.2/100,000; female: 11.2/100,000; male: 15.2/100,000), as well as mortality (total: 7.3/100,000; female: 6.2/100,000; male: 8.4/100,000) due to leukemia. Besides, the high HDI quintile had a higher number of deaths than other regions (Table 1).

In terms of MIR, Africa exhibited the greatest MIR values (both sexes: 0.75; female: 0.76; male: 0.74) when compared to the other continents. On the other hand, the MIRs were higher in the WHO South-East Asia, East Mediterranean, and Africa regions. Moreover, the low HDI region displayed the highest MIRs for females (0.74), males (0.78), and both genders (0.76). These findings suggest that the MIR values were more pronounced in less developed regions. Regarding the relative MIRs across different continents and WHO region categories, Oceania (1.08) and Americas (1.02) had the highest, while Asia (0.95), followed by the Western Pacific (0.93) and Africa (0.93) regions, demonstrated the lowest relative MIRs. As a result, the relative MIRs indicate a gender difference in leukemia (Table 1).

### Country-wise differences in the HDI, health expenditures, and in the leukemia burden

Table 2 gives the HDI, CHE per capita values, incidence, mortality, and ASR-based MIR of leukemia among 56 selected countries in the year 2020. As observed, Norway exhibited the highest HDI (0.957), whilst the lowest HDI was detected in Mauritius (0.546). Of note, the range of CHE/GDP spanned from 2.9% in Qatar and 16.7% in the United States of America.

**Table 2:** Summary of human development index, current health expenditure of incidence, mortality, and ASR-based mortality-to-incidence ratio due to leukemia among selected countries (*n* = 56)

Country	HDI		CHE		Incidence			Mortality			ASR-based MIR		
	Score	Rank	Per capita	% of GDP	ASIR	CR	Cum.Risk	ASMR	CR	Cum.Risk	2012	2020	$\delta$ MIR
Argentina	0.845	46	946	9.5	5.9	7.20	1.16	3.7	5	0.93	0.74	0.63	0.11
Australia	0.944	8	5427	9.9	10.7	17.60	2.38	3.4	7.9	1.34	0.49	0.32	0.17
Austria	0.922	18	5242	10.4	7.7	14	1.63	3.4	8.9	1.19	0.33	0.44	0.11
Belarus	0.823	53	399	5.8	7.5	11.10	1.27	3.7	7	0.95	0.61	0.49	0.12
Belgium	0.931	14	4960	10.7	11.9	21.20	2.38	3.8	10.4	1.43	0.41	0.32	0.09
Brazil	0.765	84	853	9.6	4.7	5.40	1.01	3.2	4	0.84	0.71	0.68	0.03
Bulgaria	0.816	56	697	7.1	5.2	8	0.79	3.1	7.2	0.86	0.61	0.60	0.01
Canada	0.929	16	5048	10.8	9.8	17.50	2.16	3.4	8.2	1.24	0.34	0.35	-0.01
Chile	0.851	43	1376	9.3	5.8	7	1.08	3.3	4.8	0.85	0.63	0.57	0.06
Colombia	0.767	83	495	7.7	6.2	6.60	1.13	4.1	4.8	0.91	0.65	0.66	-0.01
Costa Rica	0.810	62	921	7.3	6.4	6.90	1.10	3.6	4.6	0.89	0.67	0.56	0.11
Croatia	0.851	43	1040	6.9	6.5	12.70	1.54	4.3	11.3	1.43	0.43	0.66	-0.23
Cuba	0.783	70	986	11.3	5.3	7.60	0.94	3.2	5.4	0.75	0.65	0.60	0.05
Cyprus	0.887	33	1996	7	8	12	1.89	5.2	10.7	1.98	0.39	0.65	-0.26
Czechia	0.900	27	1844	7.8	7.4	13.90	1.69	3.5	8.7	1.18	0.46	0.47	-0.01
Denmark	0.940	10	6003	9.9	8.3	15.50	1.80	3.6	10	1.4	0.42	0.43	-0.01
Ecuador	0.759	86	486	7.8	6.7	6.80	1.24	4.8	5.1	1.05	0.73	0.71	0.02
Egypt	0.707	116	149	4.7	5.7	5.10	0.94	4.5	3.8	0.95	0.77	0.79	-0.02
Estonia	0.892	29	1599	6.7	8.7	14.80	1.63	3.8	10.3	1.3	0.51	0.44	0.07
Finland	0.938	11	4450	9.1	6.8	12.40	1.36	2.4	7.1	0.89	0.34	0.35	-0.01
France	0.901	26	4492	11.1	8.9	18	2.05	3.6	10.6	1.37	0.36	0.40	-0.04
Germany	0.947	6	5440	11.7	8.2	16.50	1.75	3.4	11.3	1.34	0.22	0.41	-0.19
Iceland	0.949	4	6275	8.6	5.9	9.70	1.39	2.3	5.6	1.03	0.30	0.39	-0.09
Ireland	0.955	2	4313	6.7	8.4	12.70	1.86	3.0	5.9	1.14	0.29	0.36	-0.07
Italy	0.892	29	2906	8.7	7.9	15.50	1.55	3.5	10.5	1.15	0.25	0.44	-0.19
Jamaica	0.734	101	327	6.1	4.7	5.30	0.80	3.6	4.1	0.69	0.69	0.76	-0.07
Japan	0.919	19	4360	10.7	6.1	10.80	0.97	2.4	7.8	0.72	0.30	0.39	-0.09
Kuwait	0.806	64	1759	5.5	6.6	4.80	1.63	4	2.8	1.24	0.60	0.61	-0.01
Latvia	0.866	37	1167	6.5	7.8	15	1.57	4.3	10.6	1.25	0.60	0.55	0.05
Lithuania	0.882	34	1370	7	9.9	19.20	2.07	3.9	10.4	1.26	0.62	0.39	0.23
Luxembourg	0.916	23	6221	5.4	6.0	12.50	2.13	2.8	6.7	1.28	0.39	0.47	-0.08
Malaysia	0.810	62	436	3.8	6.3	5.90	0.75	4.4	4.6	0.66	0.76	0.70	0.06
Malta	0.895	28	2532	8.2	5.2	9.70	1.14	1.7	5	0.7	0.32	0.33	-0.01
Mauritius	0.546	157	686	3.3	6.1	7.30	1.09	3.5	4.8	0.76	0.58	0.57	0.01
Netherlands	0.944	8	5335	10.1	7.8	13.90	1.54	3.3	9.5	1.34	0.33	0.42	-0.09
New Zealand	0.931	14	4211	9.7	8.8	14.30	1.94	4.2	9.3	1.53	0.43	0.48	-0.05
Norway	0.957	1	8007	10.5	8.7	14.50	1.83	3.1	7.1	1.1	0.35	0.36	-0.01
Philippines	0.718	107	142	4.1	5.7	5.30	1.04	4.3	4	0.87	0.65	0.75	-0.10
Poland	0.880	35	-	6.4	7.0	12	1.48	3.9	9	1.25	0.55	0.56	-0.01
Portugal	0.864	38	2221	9.5	7.4	15.20	1.61	3.4	9.5	1.1	0.38	0.46	-0.08
Qatar	0.848	45	1807	2.9	4.8	3.20	0.65	3.2	2.1	0.53	0.65	0.67	-0.02
Russian Federation	0.824	52	653	5. 7	6.5	9.40	1.09	3.3	5.6	0.74	0.59	0.51	0.08
Serbia	0.806	64	641	8. 7	7.9	12.1	1.39	4.1	8.1	1.05	0.67	0.52	0.15
Singapore	0.938	11	2633	4.1	8.9	11.1	1.55	3.1	5.2	0.92	0.37	0.35	0.02
Slovakia	0.860	39	1342	6.9	8.4	14.30	1.86	5	9.7	1.42	0.53	0.59	-0.06
Slovenia	0.917	22	2219	8.5	7.3	15.6	1.91	4	12.5	1.66	0.42	0.55	-0.13
South Africa	0.709	114	546	9.1	3.7	3.2	0.89	2.8	2.5	0.77	0.79	0.76	0.03
Spain	0.904	25	2711	9.1	6.8	12.70	1.42	2.9	8	1.01	0.29	0.43	-0.14
Sweden	0.945	7	5671	10.9	7.6	13.3	1.49	3	8.3	1.12	0.32	0.39	-0.07
Switzerland	0.955	2	9666	11.3	7.5	13.5	1.59	2.8	7.6	1.04	0.27	0.37	-0.10
Thailand	0.777	79	296	3.8	5.4	6.60	0.87	3.4	4.7	0.67	0.60	0.63	-0.03
Trinidad and Tobago	0.796	67	1168	7.0	5.6	6.3	0.77	3.7	4.8	0.69	0.71	0.66	0.05
Ukraine	0.779	74	248	7.1	6	8.3	0.85	3.2	5	0.53	0.57	0.53	0.04
United Kingdom	0.932	13	4313	10.1	8.7	16.2	2	2.9	7.8	1.14	0.38	0.33	0.05
United States of America	0.926	17	10,921	16.7	11.1	18.5	2.43	3.2	7.2	1.19	0.55	0.29	0.26
Uruguay	0.817	55	1661	9.3	6.3	9.7	1.36	3.4	6.8	1.1	0.70	0.54	0.16

*HDI* human development index, *CHE* current health expenditure, *GDP* gross domestic product, *ASIR* age-standardized incidence rate, *CR* crude rate, *Cum* cumulative, *ASMR* age-standardized mortality rate, *MIR* mortality-to-incidence ratio

**Fig. 2:**
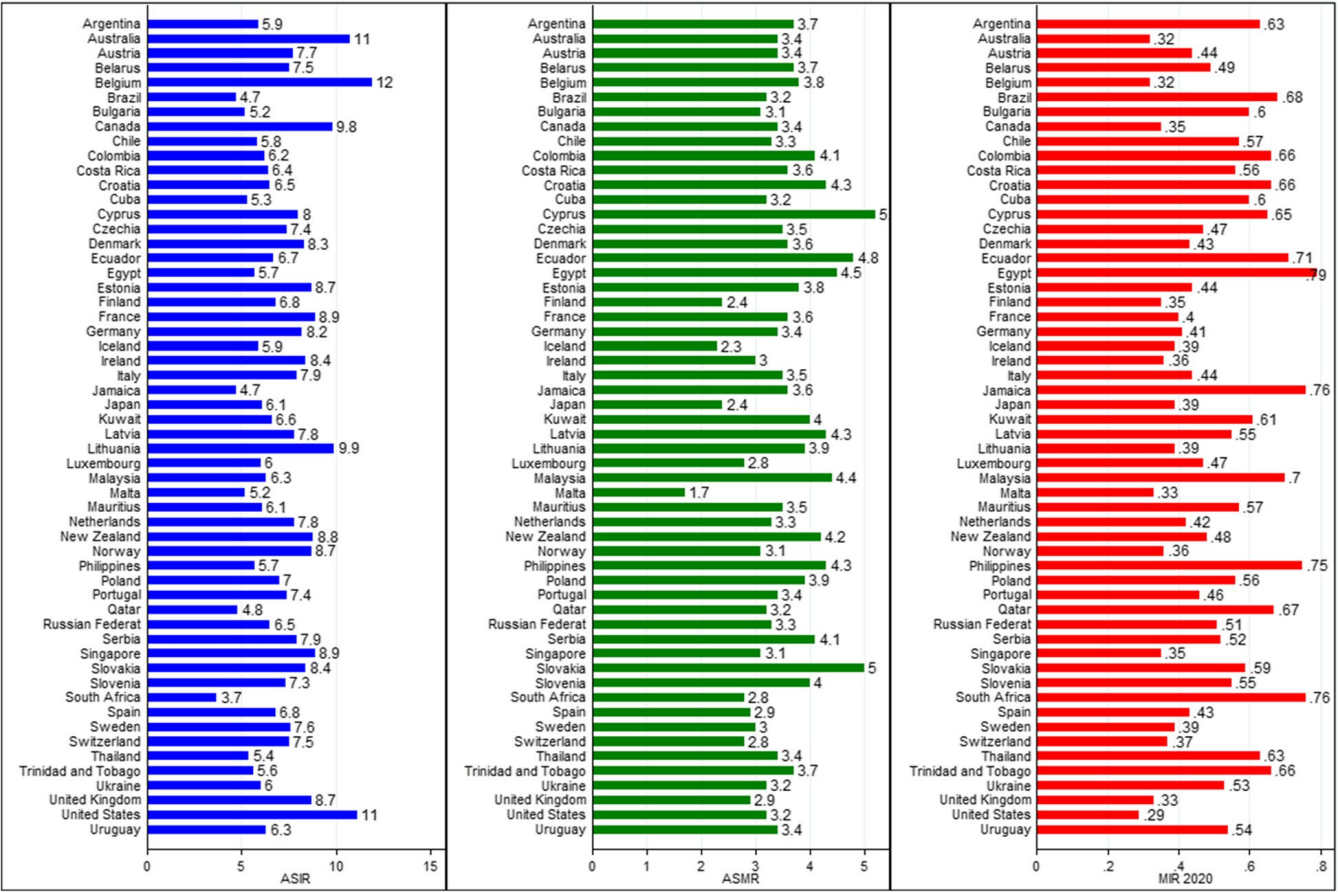
Country-wise age-standardized rates and mortality-to-incidence ratio due to leukemia in 2020

We also identified differences in leukemia outcomes across countries. It was found that Belgium (11.9/100,000) and Cyprus (5.2/100,000) had the highest ASIR and ASMR in 2020, respectively. By contrast, the lowest ASIR and ASMR were occurred in South Africa (3.7/100,000) and Malta (1.7/100,000), respectively. When considering the ASR-based MIR in 2012, South Africa (0.79) had the highest ASR-based MIR of leukemia among all included countries, while Germany (0.22) had the lowest. Furthermore, the ASR-based MIR was highest in Egypt (0.79) and lowest in the United States (0.29) in 2020 (Fig. 2). The crude rates of leukemia cancer burden are proved in Supplementary Table S1.

A comparison of ASR-based $\delta$MIR among the investigated countries revealed that the $\delta$MIRs in most countries were negative. The largest value for ASR-based $\delta$MIR was documented in the United States of America (0.26), followed by Lithuania (0.23), whilst the lowest values were obtained in Cyprus (-0.26), as well as Croatia (-0.23). Details about the gender differences in the outcome of leukemia and related ASR-based MIR, separately for females and males, are summarized in Tables 3 and 4, respectively.

**Table 3:** The age-standardized incidence and mortality rates along with the ASR -based mortality-to-incidence ratio due to leukemia among females of selected countries (*n* = 56)

Country	Incidence		Mortality		ASR-based MIR		
	ASIR	Cum.Risk	ASMR	Cum.Risk	2012	2020	δMIR
Argentina	4.9	0.89	2.9	0.7	0.71	0.59	0.12
Australia	8.4	1.77	2.6	1.02	0.42	0.31	0.11
Austria	6.2	1.25	2.6	0.92	0.28	0.42	-0.14
Belarus	6.7	1.02	3	0.76	0.62	0.45	0.17
Belgium	10.4	1.98	3.1	1.15	0.37	0.30	0.07
Brazil	4	0.81	2.7	0.68	0.66	0.67	-0.01
Bulgaria	4.6	0.68	2.3	0.69	0.56	0.50	0.06
Canada	7.6	1.58	2.6	0.92	0.31	0.34	-0.03
Chile	5.1	0.87	2.7	0.67	0.58	0.53	0.05
Colombia	5.4	0.94	3.5	0.76	0.63	0.65	-0.02
Costa Rica	5.6	0.91	3	0.72	0.62	0.53	0.09
Croatia	5	1.19	3.4	1.08	0.39	0.68	-0.29
Cuba	4.6	0.78	2.6	0.61	0.60	0.56	0.04
Cyprus	6.5	1.59	3.6	1.45	0.35	0.55	-0.20
Czechia	5.6	1.28	2.7	0.91	0.44	0.48	-0.04
Denmark	6.6	1.33	3.1	1.13	0.40	0.47	-0.07
Ecuador	5.9	1.04	4.3	0.89	0.71	0.73	-0.02
Egypt	5.1	0.87	3.9	0.87	0.75	0.76	-0.01
Estonia	6.8	1.25	2.9	0.94	0.46	0.43	0.03
Finland	5.4	1.03	2	0.68	0.31	0.37	-0.06
France	7	1.59	2.6	1.01	0.33	0.37	-0.04
Germany	6.7	1.36	2.7	1.04	0.21	0.40	-0.19
Iceland	4	0.81	1.7	0.63	0.26	0.42	-0.16
Ireland	7.4	1.46	2.5	0.91	0.25	0.34	-0.09
Italy	6.8	1.27	2.8	0.89	0.22	0.41	0.19
Jamaica	4.4	0.64	3.3	0.6	0.67	0.75	-0.08
Japan	5.4	0.78	1.7	0.51	0.25	0.31	-0.06
Kuwait	6.6	1.64	4.2	1.24	0.55	0.64	-0.09
Latvia	6.9	1.4	3.4	1.08	0.57	0.49	0.08
Lithuania	8.7	1.76	2.9	1.08	0.60	0.33	0.27
Luxembourg	3.1	1.23	1.8	0.88	0.35	0.58	-0.23
Malaysia	5.6	0.69	3.9	0.61	0.72	0.69	0.03
Malta	5	0.99	1.3	0.54	0.31	0.26	0.05
Mauritius	5.1	0.74	2.9	0.63	0.54	0.57	-0.03
Netherlands	6.3	1.16	2.5	0.97	0.30	0.39	-0.09
New Zealand	7.6	1.62	3.4	1.24	0.40	0.45	-0.05
Norway	7.2	1.46	2.4	0.8	0.30	0.33	-0.03
Philippines	5.1	0.86	3.7	0.71	0.63	0.72	-0.09
Poland	5.7	1.25	3.1	0.97	0.52	0.54	-0.02
Portugal	6.3	1.28	2.7	0.83	0.34	0.43	-0.09
Qatar	3.5	0.37	2.4	0.3	0.47	0.68	-0.21
Russian Federation	5.7	0.92	2.7	0.61	0.56	0.47	0.09
Serbia	6.6	1.1	3.3	0.81	0.64	0.50	0.14
Singapore	7.9	1.28	2.5	0.68	0.31	0.32	-0.01
Slovakia	6.5	1.5	4.4	1.26	0.50	0.68	-0.18
Slovenia	6	1.43	2.9	1.19	0.36	0.48	-0.12
South Africa	2.9	0.73	2.2	0.63	0.75	0.76	-0.01
Spain	5.6	1.07	2.2	0.77	0.25	0.39	-0.14
Sweden	6.2	1.16	2.4	0.92	0.30	0.39	-0.09
Switzerland	6	1.16	2.1	0.73	0.24	0.35	-0.11
Thailand	4.9	0.76	3	0.58	0.60	0.61	-0.01
Trinidad and Tobago	5.1	0.6	3.5	0.51	0.68	0.69	-0.01
Ukraine	4.9	0.66	2.5	0.4	0.55	0.51	0.04
United Kingdom	7	1.51	2.2	0.85	0.33	0.31	0.02
United States of America	9	1.87	2.5	0.88	0.54	0.28	0.26
Uruguay	5.1	1.07	2.7	0.84	0.66	0.53	0.13

*ASIR* age-standardized incidence rate, *Cum* cumulative, *ASMR* age-standardized mortality rate, *MIR* mortality-to-incidence ratio

**Table 4:** The age-standardized incidence and mortality rates along with the ASR -based mortality-to-incidence ratio due to leukemia among males of selected countries (*n* = 56)

Country	Incidence		Mortality		ASR-based MIR		
	ASIR	Cum.Risk	ASMR	Cum.Risk	2012	2020	$\delta$ MIR
Argentina	7.1	1.61	4.8	1.33	0.78	0.68	0.10
Australia	13.2	3.1	4.3	1.75	0.55	0.32	0.23
Austria	9.4	2.15	4.3	1.58	0.38	0.46	-0.08
Belarus	9	1.87	5	1.38	0.61	0.55	0.06
Belgium	13.6	2.92	4.7	1.83	0.45	0.34	0.11
Brazil	5.6	1.3	3.9	1.08	0.75	0.70	0.05
Bulgaria	5.9	0.97	4.9	1.26	0.65	0.83	-0.18
Canada	12.2	2.89	4.3	1.67	0.38	0.35	0.03
Chile	6.6	1.38	4	1.12	0.67	0.61	0.06
Colombia	7.1	1.37	4.8	1.1	0.69	0.68	0.01
Costa Rica	7.3	1.34	4.2	1.11	0.73	0.57	0.16
Croatia	8.3	2.15	5.6	2.11	0.49	0.67	-0.18
Cuba	6	1.13	3.8	0.93	0.69	0.63	0.06
Cyprus	9.5	2.26	7	2.7	0.45	0.74	-0.29
Czechia	9.4	2.28	4.5	1.62	0.50	0.48	0.02
Denmark	10.1	2.39	4.4	1.77	0.45	0.43	0.02
Ecuador	7.5	1.5	5.3	1.26	0.75	0.71	0.04
Egypt	6.3	1.03	5	1.04	0.78	0.79	-0.01
Estonia	11.2	2.35	5.4	2.12	0.57	0.48	0.09
Finland	8.4	1.82	3	1.2	0.39	0.36	0.03
France	11.1	2.69	4.9	1.89	0.41	0.44	-0.03
Germany	9.8	2.26	4.4	1.79	0.23	0.45	-0.22
Iceland	7.9	2.09	3.1	1.56	0.34	0.39	-0.05
Ireland	9.6	2.36	3.6	1.43	0.33	0.37	-0.04
Italy	9.2	1.91	4.4	1.52	0.28	0.48	-0.20
Jamaica	5.1	0.96	3.9	0.79	0.70	0.76	-0.06
Japan	6.8	1.24	3.1	1.03	0.35	0.45	-0.10
Kuwait	6.6	1.62	3.9	1.23	0.63	0.59	0.04
Latvia	9.1	1.89	5.5	1.6	0.64	0.60	0.04
Lithuania	11.7	2.74	5.3	1.58	0.66	0.45	0.21
Luxembourg	9.2	3.37	4	1.92	0.45	0.43	0.02
Malaysia	7	0.82	4.9	0.71	0.80	0.70	0.10
Malta	5.5	1.34	2.3	0.98	0.41	0.42	-0.01
Mauritius	7.3	1.62	4.1	0.93	0.62	0.56	0.06
Netherlands	9.5	2	4.3	1.85	0.37	0.45	-0.08
New Zealand	10.2	2.32	5.2	1.88	0.47	0.51	-0.04
Norway	10.4	2.27	3.9	1.5	0.40	0.37	0.03
Philippines	6.5	1.37	5	1.15	0.68	0.77	-0.09
Poland	8.4	1.81	5	1.75	0.60	0.59	0.01
Portugal	8.8	2.11	4.3	1.53	0.43	0.49	-0.06
Qatar	5.6	0.82	3.7	0.67	0.74	0.66	0.08
Russian Federation	7.9	1.44	4.2	1.02	0.62	0.53	0.09
Serbia	9.5	1.8	5.1	1.4	0.69	0.54	0.15
Singapore	9.9	1.92	3.9	1.23	0.42	0.39	0.03
Slovakia	10.7	2.4	5.9	1.67	0.56	0.55	0.01
Slovenia	8.9	2.69	5.5	2.41	0.48	0.62	-0.14
South Africa	4.6	1.16	3.6	1	0.86	0.78	0.08
Spain	8.2	1.89	3.8	1.34	0.33	0.46	-0.13
Sweden	9	1.89	3.6	1.37	0.35	0.40	-0.05
Switzerland	9.2	2.14	3.7	1.47	0.31	0.40	-0.09
Thailand	6.1	1.01	3.9	0.79	0.61	0.64	-0.03
Trinidad and Tobago	6.2	0.99	3.9	0.93	0.75	0.63	0.12
Ukraine	7.6	1.21	4.2	0.78	0.60	0.55	0.05
United Kingdom	10.6	2.61	3.7	1.51	0.44	0.35	0.09
United States of America	13.4	3.15	4.1	1.6	0.56	0.30	0.26
Uruguay	7.8	1.88	4.4	1.61	0.74	0.56	0.18

*ASIR* age-standardized incidence rate, *Cum* cumulative, *ASMR* age-standardized mortality rate, *MIR* mortality-to-incidence ratio

### The gender differences in the associations of leukemia MIR and δMIR with the HDI, the CHE/GDP, and the CHE per capita among various countries

**Fig. 3:**
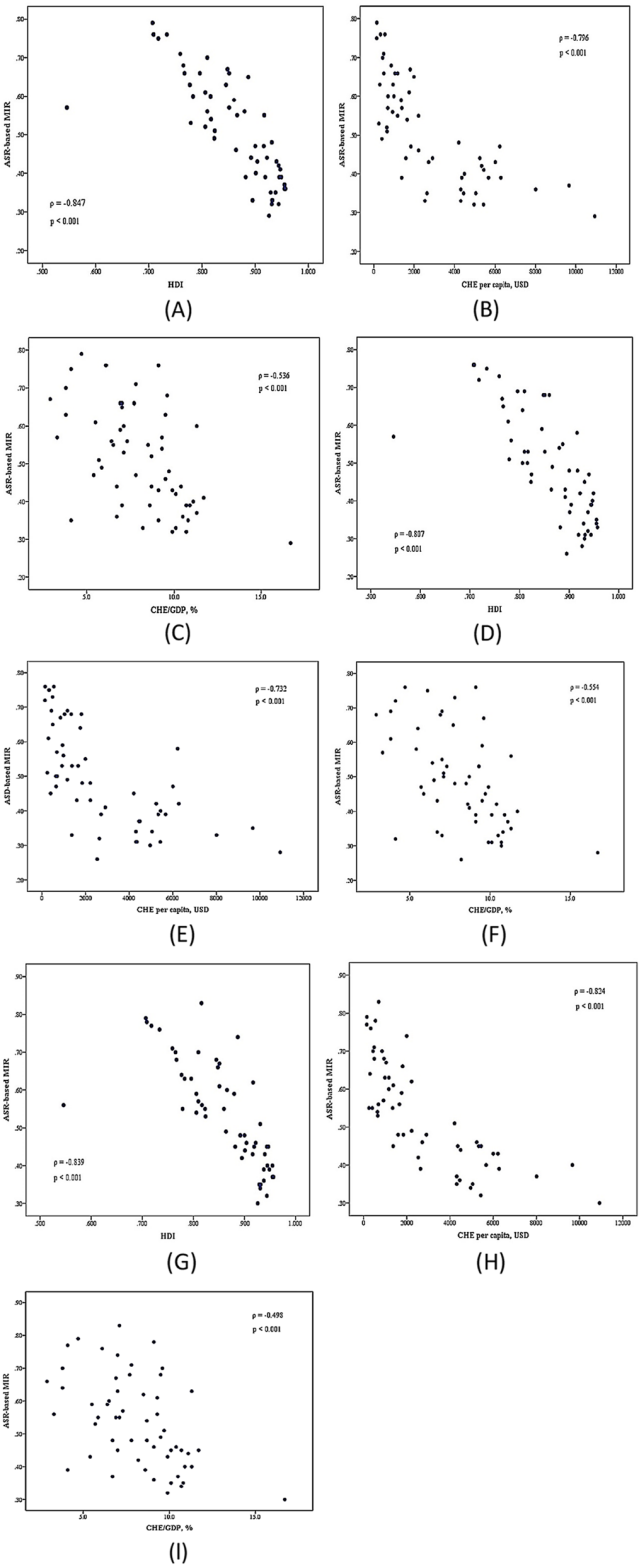
The correlations of the age-standardized rate (ASR)-based mortality-to-incidence ratio (MIR), the human development index, the current health expenditure per capita, and current health expenditure as a percentage of gross domestic product with leukemia in both genders (**A** to **C**), females (**D** to **F**), and males (**G** to **I**) in 2020, across all selected countries

To assess healthcare disparities, bivariate correlations were investigated between the ASR-based MIR and HDI, CHE per capita, and CHE/GDP. As observed, the ASR-based MIR of leukemia had a significant negative correlation with all three indicators for both genders ($\rho$= -0.847, *ρ* = -0.796, and *ρ* = -0.538, respectively), females ($\rho$= -0.807, $\rho$= -0.732, and *ρ* = -0.554, respectively), and males ($\rho$= -0.839, *ρ* = -0.824, and *ρ* = -0.498, respectively) in 2020 (all *p* < 0.001; Fig. 3A-I). Notably, countries with higher HDI values, higher CHE per capita values, and a greater CHE/GDP percentage tended to have a lesser ASR-based MIR. Meanwhile, significant negative correlations were detected between CR-based MIR, HDI (*ρ* = -0.566, *p* < 0.001), CHE per capita (*ρ* = -0.540, *p* < 0.001), and CHE/GDP ($\rho$= -0.355, *p* = 0.012) among females. Additionally, the CR-based MIR exhibited negative correlations with higher HDI, CHE per capita, and CHE/GDP values for males (Supplementary Fig. S1A-I).

**Fig. 4:**
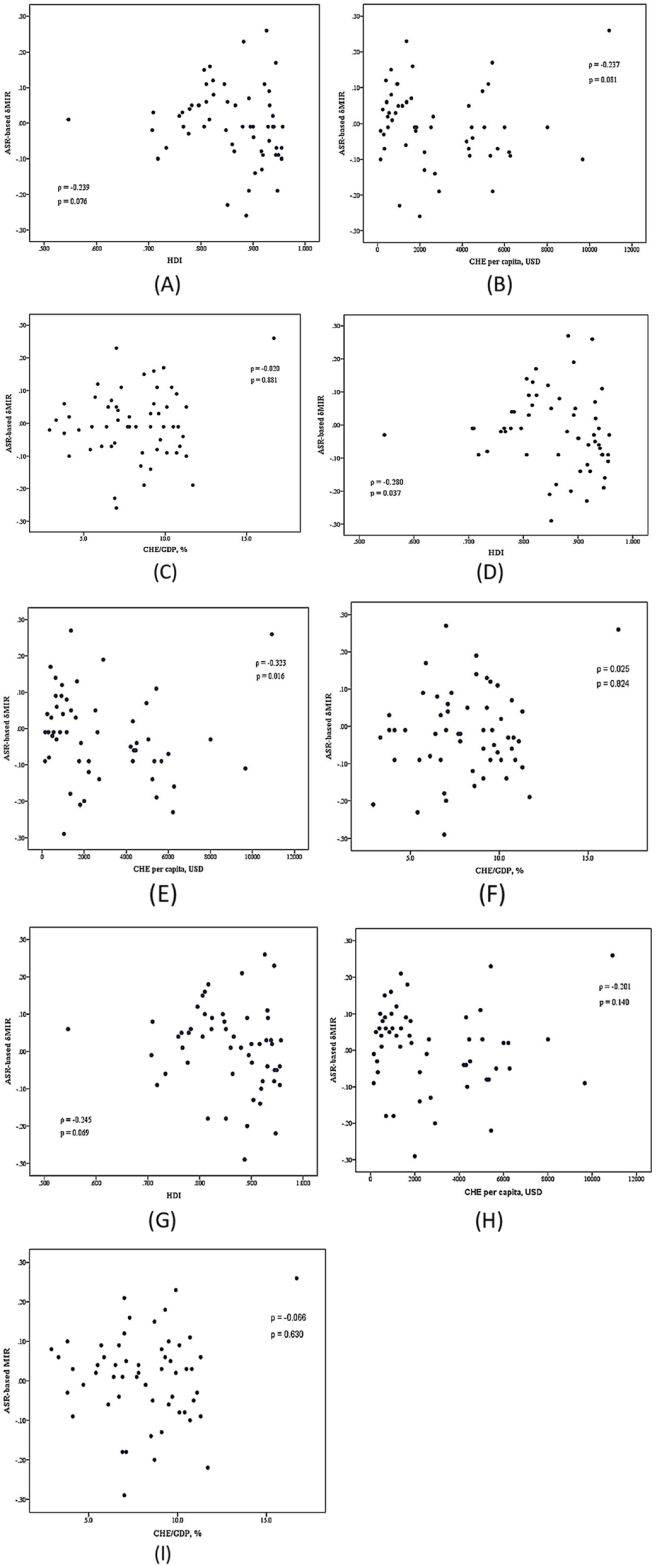
The correlations of the ASR-based δmortality-to-incidence ratio (δMIR), the human development index, the current health expenditure per capita, and current health expenditure as a percentage of gross domestic product with leukemia in both genders (**A** to **C**), females (**D** to **F**), and males (**G** to **I**)  in 2020, across all selected countries

We further utilized the ASR-based δMIR as an innovative parameter to elucidate improved outcomes in leukemia. For both genders, the HDI and CHE per capita revealed negative correlations with the ASR-based δMIR (*ρ* = -0.239, *p* = 0.076; *ρ* = -0.237, *p* = 0.081, respectively), while the CHE/GDP showed a positive association with the ASR-based δMIR (*ρ* = 0.020, *p* = 0.881) (Fig. 4A-C). Among female patients, the ASR-based δMIR was significantly and negatively correlated with the HDI and CHE per capita (*ρ* = -0.280, *p* = 0.037; *ρ* = -0.323, *p* = 0.016, respectively). However, the ASR-based δMIR failed to indicate any significant correlation with the CHE/GDP percentage (*ρ* = 0.025, *p* = 0.854) (Fig. 4D-F). On the other hand, the HDI, the CHE per capita, and the CHE/GDP percentage showed trends toward negative correlations with the ASR-based δMIR among males with leukemia, but  these trends were not statistically significant (*ρ* = -0.245, *p* = 0.069; *ρ* = -0.201, *p* = 0.140; *ρ* = -0.066, *p* = 0.630, respectively) (Fig. 4G-I). Moreover, it was found that the correlations between the CR-based δMIR and all three indicators were negative for both genders (Supplementary Fig. S2A-I).

## Discussion

This study examined the global burden of leukemia and the associations between MIR, δMIR and HDI, CHE per capita, and CHE/GDP ratio across countries. The estimates of leukemia incidence and mortality were procured from one of the most reliable sources, the GLOBOCAN 2020 database, focusing on 56 countries with quality data from cancer registries. We observed 474,519 new cases and 311,594 deaths from leukemia in 2020, ranking it among the top-10 leading cancers worldwide. Although the highest absolute burden (leukemia incidence and deaths) was detected in Asia, the highest incidence rates were found in Northern America, with the lowest rates in African regions. As per the country’s development levels, the highest incidence rates were observed in high and very high HDI countries, whereas MIR was the lowest in high and very high HDI countries. According to our correlation analysis, MIR was negatively correlated with HDI, CHE per capita, and CHE/GDP ratio, implying that the countries with higher HDI and health expenditures had lower MIR (i.e., higher survival rates) than lesser developed countries.

We found that the incidence and mortality related to leukemia were heterogeneous from country to country. Specifically, Belgium and United States of America had the highest ASIR, whereas Cyprus had the highest ASMR in 2020. The differences in leukemia burden across countries and the shifting patterns over time not only reflect the success of past prevention methods, but also suggest the implementation of new and customized prevention approaches. Similarly, previous studies have exhibited higher, although decreasing, incidence rates of leukemia in countries of America and Europe [26]. The downward trend in leukemia incidence in some areas may have been at least partly driven by reducing the exposure to environmental risk factors, abstaining from high-risk parental behaviours, increasing the intake of folate and vitamin supplementation, and expanding the genetic screening for high-risk germline mutations. On the other hand, continuous improvement of healthcare facilities and the quality of cancer surveillance could have influenced the increasing pattern of incidence in most countries [1, 26, 27]. Further, as the burden of the majority of leukemia types increases with age, the higher life expectancy and aging population in the developed countries might partially explain greater incidence rates [28]. Older individuals experience a more unfavourable outlook in terms of their health condition, as they carry a greater burden of illness and struggle to endure the toxic effects of chemotherapy [29]. Smoking has been identified as a primary risk factor for certain leukemia types. The higher smoking prevalence in Europe and parts of America can also partially explain higher incidence rates in these countries [30]. In a number of researches, it has been highlighted that myeloid cells can suffer damage from smoking-related carcinogens such as benzene, 1–3 butadiene and formaldehyde [31–33]. Not only active smoking, but paternal smoking is also linked with certain childhood leukemia types [34]. Overall, examining the exposures thoroughly in different nations could unveil further evidence regarding the factors contributing to leukemia.

Our study has also identified that male population had a greater incidence and mortality of leukemia than those of females worldwide in terms of both absolute counts and rates per 100,000 persons. This finding is in concordance with a prior work at the global level [4], as well as in individual countries such as the United States [35, 36]. The higher burden among males of a few leukemia types might be attributed to the greater prevalence of risk factors such as smoking and occupational carcinogens [37, 38]. A study has suggested some protective effects of estrogen in females [39] and male–female differences might be smaller during childhood as estrogen/androgen levels are low during childhood [40]. Although a number of factors such as genetic polymorphisms, epigenetics, hormones, senescence, immunity, and angiogenesis may be part of the explanation of the gender differences, the causes of difference between males and females in the burden of disease are not yet established [14, 41].

We further found that CHE per capita and CHE/GDP were negatively correlated with MIR, indicating that the countries incurring higher expenditures, on average, have better disease outcomes (reflected by low MIR) than countries with lower health expenditures. It supports that cancer treatment is still costly and resource-intensive, requiring both availability of health infrastructure and affordability of cancer treatment. In low-resource countries, health infrastructure is scarce; due to the lack of universal health coverage or health insurance, people cannot afford the costly cancer treatments even if cancer infrastructure is put into place [42]. Studies conducted in low- and middle-income countries (LMICs) such as China [43, 44] and Bangladesh [45], found that the treatment cost of acute lymphoblastic leukemia is lower than in high-income countries (HICs) [46]; but still higher in relation to per capita income in these countries. Because of costly treatments, therapy abandonment of childhood cancers, including leukemia, is a well-known hindrance, while improving disease outcomes in LMICs [47, 48]. In terms of childhood leukemia, such as acute lymphoblastic leukemia (ALL), the event-free survival rate has reached as high as 90% in HICs, whereas in low and middle-income countries, the survival rates are still dismal due to therapy abandonment, delay in diagnosis, lack of supportive care, and treatment failure [49, 50]. In the case of acute leukemia, perhaps 12–13% of patients was likely to abandon the treatment, most may due to high out-of-pocket expenditures, transportation costs, and economic hardship resulting from the treatments [51]. The dismal disease outcomes, reflected by high MIR, in countries with low CHE per capita and CHE/GDP is also apparent from scarce cancer care infrastructure in these countries. Leukemia diagnosis and classification require multiple testing relating to blood testing, chromosome test (cytogenetics, Fluorescence In Situ Hybridization or polymerase chain reaction), bone marrow aspiration and biopsy, flow cytometry, and immunohistochemical (IHC) analysis [11, 52]. However, several cases of leukemia are either misdiagnosed, underdiagnosed, or due to inadequate histopathological confirmation of specific malignancies, and appropriate treatments are not offered to some patients in low-resource countries.

Imatinib, a tyrosine Kinase inhibitor, has tremendously boosted the survival rates of certain leukemia types for patients treated with Imatinib [53–55]. BCR-ABL testing, used to diagnose the Philadelphia chromosome, might be limited or not cost-effective in certain low-resource countries. Imatinib is provided free of cost through the Glivec International Patient Assistance Program (GIPAP) [56]. However, if leukemia is misdiagnosed or diagnosed as another leukemia type, proper treatment might not be offered, even if it is available free of cost. Moreover, flow cytometry, blood film examination, or bone marrow evaluation are typically available at tertiary centers. Thus, referrals from primary or secondary centers to tertiary centers might result in delays in diagnosis and treatment, ultimately leading to a poorer prognosis. 

By 2040, in the global context, the burden of leukemia is projected to increase from 474,519 to 647,333 cases, with the most significant growth (73.2%) anticipated in Africa. These projections are based on the assumption that the 2020 incidence rate will persist in 2040, implying that risk factors will remain unchanged. However, we contend that if diagnoses are improved, along with enhancements in cancer registration throughout Africa, the actual number in 2040 could exceed the prediction by Sung et al. in 2021 [2]. Consequently, prioritizing capacity building in terms of healthcare infrastructure (including hospitals, diagnostic equipment, and personnel) and cancer registries becomes imperative for policymakers in the region. 

As described earlier, since the calculation of MIR is based on the ratio of the CR of mortality to the CR of incidence [57], the potential effect of varied age groups across countries was not accounted for. Hence, we employed an ASR-based MIR, as well as ASR-based δMIR to examine gender differences in leukemia. In the current article, it was found that there were differences in relationship between CR-based MIR and the HDI, the CHE per capita, and the CHE/GDP in females and males as with the CR-based δMIR. These findings provide evidence for the impact of heterogeneous age and gender groups as confounding factors across countries in relation to leukemia.

Possible limitations of our study should be pointed out. The primary constraint in this literature pertains to the scarcity of high-quality data from cancer registries in several LMICs, which hindered our ability to analyze all 185 countries. The GLOBOCAN estimates heavily depend on data from cancer registries; however, in some LMICs, the absence of cancer registries and inadequate cancer infrastructure (e.g., diagnostic facilities, medical professionals, and nursing staff) may have led to underreporting of cases or deaths. Consequently, the GLOBOCAN estimates for incidence and mortality could be underestimated in these regions. For example, countries like Burundi face challenges of underfunded cancer registries and a lack of dedicated personnel, underscoring the critical need for adequate funding to ensure comprehensive and accurate cancer data collection in such nations. Secondly, GLOBOCAN does not provide leukemia data disaggregated by etiology, such as chronic vs. acute and lymphoid vs. myeloid. A disaggregated analysis of the main leukemia types would have offered a much more comprehensive examination of the global burden of leukemia and could assist countries in formulating targeted policies. Thirdly, this is an observational study, and no causal statements can be derived from the results of our study. Nonetheless, they can serve as a basis for generating hypotheses in the future. Lastly, because of the cross-sectional nature of the database available from GLOBOCAN, assessing the trends in leukemia burden was not feasible.

## Conclusions

In summary, our findings clearly delineated gender variation in ASR-based MIR and ASR-based δMIR across 56 countries. However, these variations can be partially attributed to social, behavioral, and biological factors. It is vital to comprehend these variances as a crucial prerequisite for the timely identification and implementation of pre-emptive measures to avert leukemia. It is also interesting to note that we observed high incidence rates in developed regions and countries, whereas the MIR was the lowest in developed countries, implying better leukemia survival rates. Leukemia diagnosis based on immunophenotype has critical implications for treatment and prognostics. However, in countries such as Burundi and the majority of SSA countries, neither flow cytometry nor IHC is available. Importantly, flow cytometry is widely used for immunophenotyping, which is essential for the accurate diagnosis and risk-directed therapy of patients with leukemia. Furthermore, IHC plays a significant role in lineage specification in leukemia. Therefore, failing to use IHC may lead to poor leukemia outcomes in such countries. Since the diagnosis and treatment of leukemia can be expensive, CHE and CHE/GDP exhibit a negative relationship with MIR. This suggests that countries with higher health expenditures tend to achieve better disease outcomes compared to low-resource countries. Thereby, improving disease outcomes for conditions like leukemia, as well as addressing other cancer-related demands, necessitates increased public health spending on cancer infrastructure. Given potential resource constraints in certain countries, it is imperative that additional donor funding be allocated towards enhancing diagnostic facilities, implementing multi-modal treatments, and establishing robust data collection systems in LMICs.

### Supplementary Information

**Additional file 1.** 

## Data Availability

The datasets analyzed during the current study are publicly available in http://gco.iarc.fr/ and https://www.who.int/gho/publications/world_health_statistics/en/

## Abbreviations

ASIR: Age-Standardized Incidence Rate
ASMR: Age-Standardized Mortality Rate
MIR: Mortality-to-Incidence Ratio
GBD: Global Burden of Disease
HDI: Human Development Index
CHE: Current Health Expenditure
CHE/GDP: CHE as a Percentage of Gross Domestic Product
δMIR: delta-mortality-to-incidence ratio
WHO: World Health Organization
CR: Crude Rate
LMICs: Low- and Middle-Income Countries
